# Association Analyses of *TP53* Mutation With Prognosis, Tumor Mutational Burden, and Immunological Features in Acute Myeloid Leukemia

**DOI:** 10.3389/fimmu.2021.717527

**Published:** 2021-10-21

**Authors:** Xiang-mei Wen, Zi-jun Xu, Ye Jin, Pei-hui Xia, Ji-chun Ma, Wei Qian, Jiang Lin, Jun Qian

**Affiliations:** ^1^ Laboratory Center, Affiliated People’s Hospital of Jiangsu University, Zhenjiang, China; ^2^ Zhenjiang Clinical Research Center of Hematology, Affiliated People’s Hospital of Jiangsu University, Zhenjiang, China; ^3^ The Key Lab of Precision Diagnosis and Treatment in Hematologic Malignancies of Zhenjiang City, Affiliated People’s Hospital of Jiangsu University, Zhenjiang, China; ^4^ Department of Hematology, Affiliated People’s Hospital of Jiangsu University, Zhenjiang, China; ^5^ Department of Otolaryngology-Head and Neck Surgery, Affiliated People’s Hospital of Jiangsu University, Zhenjiang, China

**Keywords:** mutation, *TP53*, prognosis, tumor mutational burden, tumor-infiltrating immune cells, acute myeloid leukemia

## Abstract

Acute myeloid leukemia (AML) is a heterogeneous disease related to a broad spectrum of molecular alterations. The successes of immunotherapies treating solid tumors and a deeper understanding of the immune systems of patients with hematologic malignancies have promoted the development of immunotherapies for the treatment of AML. And high tumor mutational burden (TMB) is an emerging predictive biomarker for response to immunotherapy. However, the association of gene mutation in AML with TMB and immunological features still has not been clearly elucidated. In our study, based on The Cancer Genome Atlas (TCGA) and BeatAML cohorts, 20 frequently mutated genes were found to be covered by both datasets in AML. And *TP53* mutation was associated with a poor prognosis, and its mutation displayed exclusiveness with other common mutated genes in both datasets. Moreover, *TP53* mutation correlated with TMB and the immune microenvironment. Gene Set Enrichment Analysis (GSEA) showed that *TP53* mutation upregulated signaling pathways involved in the immune system. In summary, *TP53* mutation is frequently mutated in AML, and its mutation is associated with dismal outcome, TMB, and immunological features, which may serve as a biomarker to predict immune response in AML.

## Introduction

Immunotherapy of cancers has opened a new era in cancer treatment. A great quantity of immunomodulatory strategies has gradually been explored in the past decade such as engineered viruses and cells with enhanced functionalities, immune checkpoint inhibitors (ICIs), and so on (1). And ICIs are changing the treatment paradigms of many tumor types (2–4). The most well-depicted inhibitory immune checkpoint is the relationship of programmed death 1 (PD-1) receptor with PD-ligand 1 (PD-L1), where clinical activity was observed in multiple solid tumor types (5).

At present, several clinical trials using anti-PD-1 or PD-L1 antibodies treat patients with acute myeloid leukemia (AML) (6, 7). It is suggested that PD-1/PD-L1 blockade may be a revolutionary immunotherapeutic strategy for AML. However, clinical trials showed that the clinical response to PD-1/PD-L1 blockade varied in different AML patients (8, 9). It is thought that factors other than PD-1 and PD-L1 may aggravate their immunosuppression and affect their effects on immunotherapy of AML patients (8, 10). Additionally, ICIs can induce immune-related adverse events that can lead to fulminant and even fatal consequences and limit applications of ICIs in many patients (11). Therefore, identifying predictive biomarkers of both efficacy and toxicity connected with the use of ICIs would greatly help guide treatment decisions. Accurate identification of patients with tumors likely to respond to immunotherapy is crucial. High tumor mutational burden (TMB) is a sensitive biomarker for response to immunotherapy largely because tumor mutations provide more opportunities to generate immunogenic neoantigens, and neoantigens enable highly specific and effective anticancer immune responses that provide an exceptionally absorbing target for immunotherapy (12).

In AML, gene mutations devoted to disease features, survival, treatment response, and promising examples demonstrate that some of those mutations might be thought to be therapeutic targets (6, 13). A number of potential shared neoantigens have been recognized for hematologic malignancies, most of which originated from well-established mutations and fusions (14). The neoantigens derived from driver gene mutations are less likely to cause immune evasion since leukemic cells have to definitely express the critical driver mutated protein to maintain their malignant phenotype (14). *TP53* aberrations have been addressed to modulate both the immune and inflammatory responses in malignant tumors, involving the regulatory T cell (Treg) recruitment and T-cell differentiation (15–17). Mutations in the *TP53* gene are strongly enriched in complex karyotype AML and associated with adverse outcomes and treatment selection in AML (18, 19). It has been reported that *TP53* mutations are related to increased leukocyte infiltration across 30 diverse cancer types, and higher TMB and higher proportions of PD-L1–expressing CD8+ T cells correlate with beneficial responses to pembrolizumab immunotherapy in patients with *TP53*-mutated lung adenocarcinoma (20, 21). However, little research has been done on the association of *TP53* gene mutations with TMB and immune response in AML.

In our study, we first found somatic mutations in AML patients using The Cancer Genome Atlas (TCGA) dataset and BeatAML dataset and then identified the common mutant genes in the two cohorts. Furthermore, the association of these gene mutations with TMB and prognosis was investigated. Finally, we explored whether *TP53* mutation was related to immune response.

## Materials and Methods

### Data Acquisition

Somatic gene mutations with clinical data were obtained from TCGA portal (https://cancergenome.nih.gov/) (n = 164). Meanwhile, BeatAML mutation, clinical, and sample annotation data were downloaded from source data (from **Table S3**) (22), and 558 cases were included in the analysis.

## Bioinformatics Analyses

Circos plot was acquired by the use of Circos (http://circos.ca/). And somatic interactions, oncoplot, and Lollipop plot were performed by the “maftools” package. Somatic interaction function that performed pairwise Fisher’s exact test was applied to survey mutually exclusive or co-occurring sets of genes. Lollipop plots were drawn for *TP53* mutation. Then, Mutation Annotation Format data files based on TCGA and BeatAML cohorts were functioned by “maftools” package to extract detailed mutational information. *TP53* mutation in AML was also evaluated by the cbioportal dataset (https://www.cbioportal.org/). Gene Set Enrichment Analysis (GSEA) (http://www.gsea-msigdb.org/gsea/index.jsp) was used based on analysis of Hallmark gene sets and curated gene sets.

### Assessment of Tumor Mutational Burden

TMB, defined as the number of somatic non-synonymous variations, which included nonsense mutation, deletion, missense mutation, splice site, and insertion. The corresponding TMB value was acquired by calculating the number of tumor mutations per Mb in each sample, and the relationship between gene mutations and TMB was visualized using the ggplot2 package.

### Tumor-Infiltrating Lymphocyte Cell Analysis

For TCGA and BeatAML datasets, the edgeR voom algorithm was used to convert the RNA sequencing data and the count data to values that were closer to the microarray results (23). CIBERSORT algorithm (24), a deconvolution approach that evaluates the proportions of 22 tumor-infiltrating lymphocyte cells (TILs) in a bulk tumor transcriptome, was used to examine the relative abundance of immune cell infiltration in different *TP53* mutation statuses. And the 22 cell types included B cells, T cells, natural killer (NK) cells, macrophages, dendritic cells, and myeloid subsets. Difference in immune cell abundance between wild group and mutation group was analyzed using a violin map. TIDE, a novel computational method that can evaluate the potential of tumor immune escape, was used to calculate immune measures (http://tide.dfcl.harvard.edu/).

### Statistical Analyses

Statistical analyses in this study were performed with R software version 4.0.4 (https://www.r-project.org/). The data were shown as either boxplots or violin plots using the ggplot2 package concerning comparison of two continuous variables, and the association between two categorical variables was evaluated by chi-square test and Fisher’s exact test. Univariate Cox regression was used to test whether gene mutations had prognostic value in AML. The prognostic effects of gene mutations were verified through Kaplan–Meier survival analysis using a log-rank test. We employed the survival package to generate Kaplan–Meier survival curves. The correlation between mutant genes and TMB was examined by Wilcoxon rank sum test. The TMB value of each patient was calculated with the TMB function of the maftools package. The two-sided with a significance level of 0.05 in all comparisons was defined as statistically significant.

## Results

### Somatic Genomic Mutations in Acute Myeloid Leukemia

**Figure 1:**
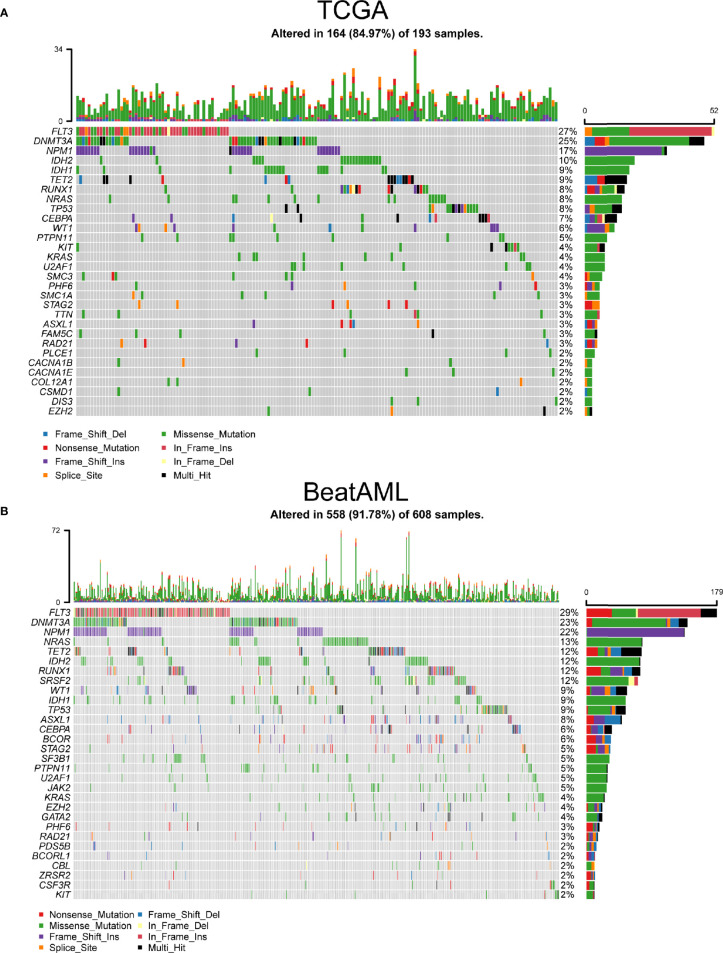
The most frequently mutated genes in acute myeloid leukemia (AML). **(A)** Waterfall plot of the frequently mutated genes in AML from The Cancer Genome Atlas (TCGA) cohort. **(B)** Oncoplot visualizing the frequently mutated genes in AML from BeatAML cohort. In the above two figures, top 30 mutated genes were ordered according to reducing mutation frequency from top to bottom. The upper panel presented mutation frequencies of genes. The bottom panel showed different gene mutation types.

Using TCGA and BeatAML cohorts, we revealed the key visualizations generated using maftools. We recognized 30 frequently mutated genes from TCGA cohort, and the 10 most frequently mutated genes were *FLT3* (27%), *DNMT3A* (25%), *NPM1* (17%), *IDH2* (10%), *IDH1* (9%), *TET2* (9%), *RUNX1* (8%), *TP53* (8%), *NRAS* (8%), and *CEBPA* (7%) (**Figure 1A**). At the same time, we also defined 30 frequently mutated genes from BeatAML cohort (**Figure 1B**), and the 10 most frequent mutations were *FLT3* (29%), *DNMT3A* (23%), *NPM1* (22%), *NRAS* (13%), *TET2* (12%), *RUNX1* (12%), *IDH2* (12%), *SRSF2* (12%), *WT1* (9%), and *TP53* (9%). Interestingly, there were 20 genes mutated in both TCGA and BeatAML cohorts of AML (**Figure S1**). Then, we analyzed these commonly mutated genes in the next study.

### Co-Occurrence of Genetic Alterations in Acute Myeloid Leukemia

**Figure 2:**
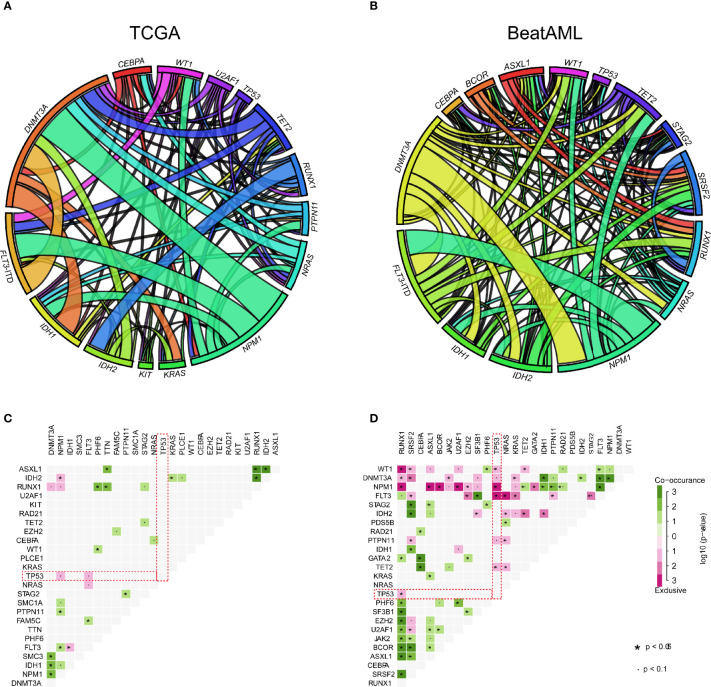
The relationship between common mutant genes in acute myeloid leukemia (AML). **(A, B)** Circos plot diagram showing the frequency of pairwise co-occurring gene mutations in AML from The Cancer Genome Atlas (TCGA) **(A)** and BeatAML **(B)** cohorts. The thickness of connecting lines between two genes denoted proportion of the number of such cases. **(C, D)** Gene pairs with co-occurrence or exclusiveness in their mutation pattern were illustrated as a triangular matrix in AML from TCGA **(C)** and BeatAML **(D)**. Green displayed tendency toward co-occurrence, whereas pink showed exclusiveness.

As shown in **Figures 2A, B**, the length of the arc indicated the frequency of gene mutations, whereas the width of the connecting lines represented the frequency of co-occurrence between two genes. The frequency of co-mutation of *NMP1* with *DNMT3A* and *FLT3-ITD* was significantly higher. In addition, we identified potentially altered gene sets that showed co-occurrence or exclusiveness in their mutation pattern. Notably, the results indicated that only *TP53* and other common mutated genes were mutually exclusive in both TCGA and BeatAML cohorts (**Figures 2C, D**).

### Prognosis of *TP53* Mutations in Acute Myeloid Leukemia

**Figure 3:**
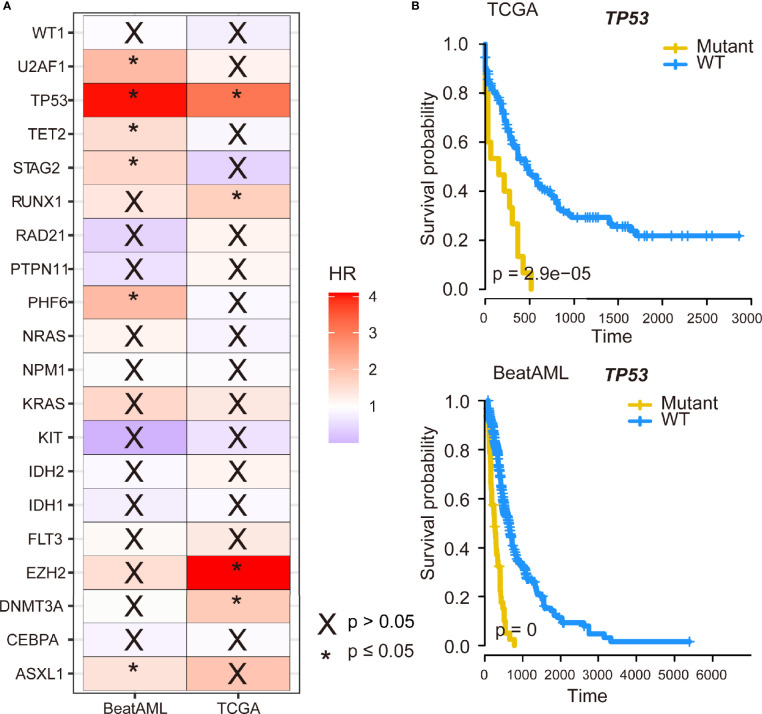
The impact of *TP53* mutation on survival of acute myeloid leukemia (AML) patients. **(A)** The 20 genes with the highest mutation frequency shared by The Cancer Genome Atlas (TCGA) and BeatAML datasets were subjected to Cox regression analysis. **(B)** Kaplan–Meier survival curves of *TP53* mutation on survival in AML.

Next, the prognostic effects of the commonly mutated genes were examined by Cox regression. As displayed in **Figure 3A**, patients with *U2AF1*, *TP53*, *TET2*, *STAG2*, *PHF6*, or *ASXL1* gene mutations had poor prognosis in BeatAML cohort and so do patients with *TP53*, *RUNX1*, *EZH2*, or *DNMT3A* mutations in TCGA cohort. Remarkably, we identified that *TP53* gene mutations were dramatically associated with worse overall survival (OS) in both TCGA and BeatAML cohorts [p ≤ 0.05, hazard ratio (HR) >1] (**Figure 3A**). Furthermore, we performed Kaplan–Meier survival analysis to validate the relationship between mutated genes and the prognosis of patients with AML. We also observed the parallel results; as shown in **Figure 3B**, *TP53* mutation was associated with a poor prognosis (p < 0.001 and p = 0). Meanwhile, *DNMT3A* and *RUNX1* gene mutations predicted poor survival probability (p = 0.001, and p = 0.042) in TCGA cohort, and patients with *TET2* and *U2AF1* gene mutations had shorter survival time (p = 0.006 and p = 0.001, respectively) in the BeatAML cohort using Kaplan–Meier methods (**Figure S2**).

### *TP53* Mutations Are Associated With Tumor Mutational Burden

**Figure 4:**
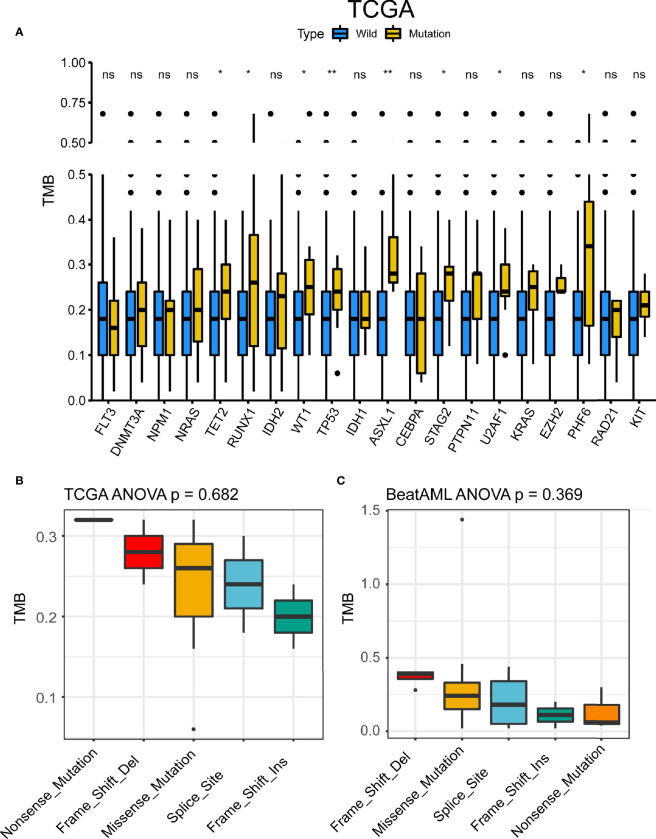
*TP53* mutation was associated with tumor mutational burden (TMB). **(A)** TMB differences between the mutant group and the wild-type group of the 20 genes with the highest mutation frequency shared by the two datasets in The Cancer Genome Atlas (TCGA). *p < 0.05, **p < 0.01, ns, not significant. **(B, C)** TMB differences of *TP53* mutant subtypes in TCGA **(B)** and BeatAML **(C)**.

We compared TMB data from public databases (TCGA and BeatAML). Among commonly mutated genes, patients with mutation in *TET2*, *RUNX1*, *WT1*, *TP53*, *ASXL1*, *STAG2*, *U2AF1*, and *PHF6* displayed significantly higher TMB levels in TCGA cohort (**Figure 4A**). There were five types of *TP53* mutations such as frame shift deletion, missense mutation, splice site, frame shift insertion, and nonsense mutation. To address the different roles of *TP53* mutation subtypes in TMB, we then detected the association between different *TP53* mutation subtypes and TMB. No difference in TMB was observed between *TP53* mutation subtypes in both TCGA and BeatAML cohorts (p = 0.682 and p = 0.369, respectively) (**Figures 4B, C**). The TMB score of AML ranged from 0.00 to 0.68 mutation/per Mb with a median of 0.18 mutation/per Mb in TCGA cohort (**Figure S3A**), and the TMB score is ranging from 0.02 to 1.44 per Mb with a median of 0.17 per Mb in BeatAML cohort (**Figure S3B**). Also, patients with mutation in *TET2*, *RUNX1*, *IDH2*, *ASXL1*, *U2AF1*, *EZH2*, *PHF6*, and *RAD21* had higher TMB levels in BeatAML cohort (**Figure S3C**). We investigated the association of TMB with *NPM1* and *FLT3*, which were mutually exclusive genes of *TP53*, and found that *NPM1* mutation subtypes were significantly connected with TMB in BeatAML (p < 0.001) (**Figure S4**).

### *TP53* Mutations in Acute Myeloid Leukemia

**Figure 5:**
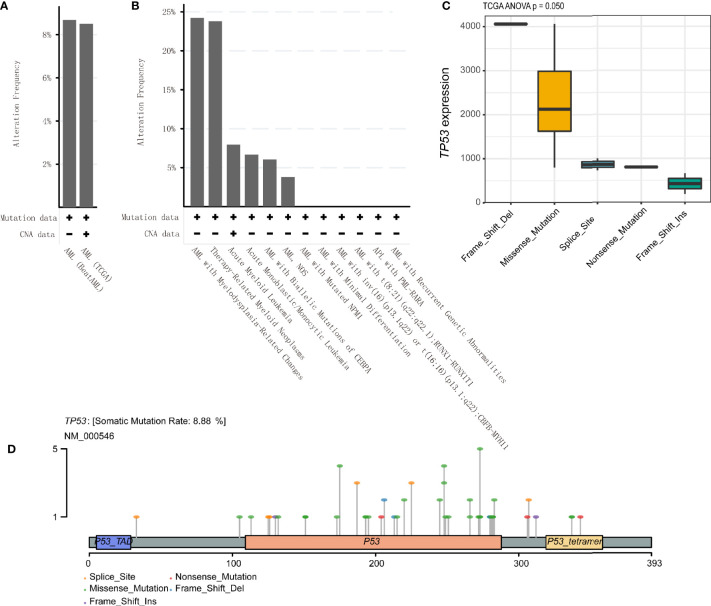
*TP53* alteration frequencies and location in acute myeloid leukemia (AML). **(A, B)** *TP53* alteration frequencies in AML were shown. **(C)** Correlation of *TP53* mutation status with *TP53* mRNA expression level in AML patients based on the analysis of The Cancer Genome Atlas (TCGA) database. **(D)** Lollipop plot indicating location and type of *TP53* mutation in AML. Circles representing individual mutations had distinct colored markings for different types of mutations.

The alteration frequencies of AML were 8.68% and 8.5% in BeatAML and TCGA cohort, respectively (**Figure 5A**). The patients of AML with myelodysplasia-related changes and therapy-related myeloid neoplasms had higher mutation rates (24.22% and 23.81%) (**Figure 5B**). Meanwhile, we compared *TP53* mutant subtypes and the *TP53* expression level, the results demonstrated that *TP53* mRNA expression was related to *TP53* mutant subtypes in TCGA (p = 0.050) (**Figure 5C**), while no differences were detected between each *TP53* group and the *TP53* expression level in BeatAML (p = 0.201) (**Figure S5**). **Figure 5D** exhibited Lollipop plot, generated by maftools, which showed mutation distribution for *TP53* in AML. And *TP53* mutations mainly included missense mutation that exhibited a high proportion.

### *TP53* Mutations Are Associated With Tumor-Infiltrating Immune Cells

**Figure 6:**
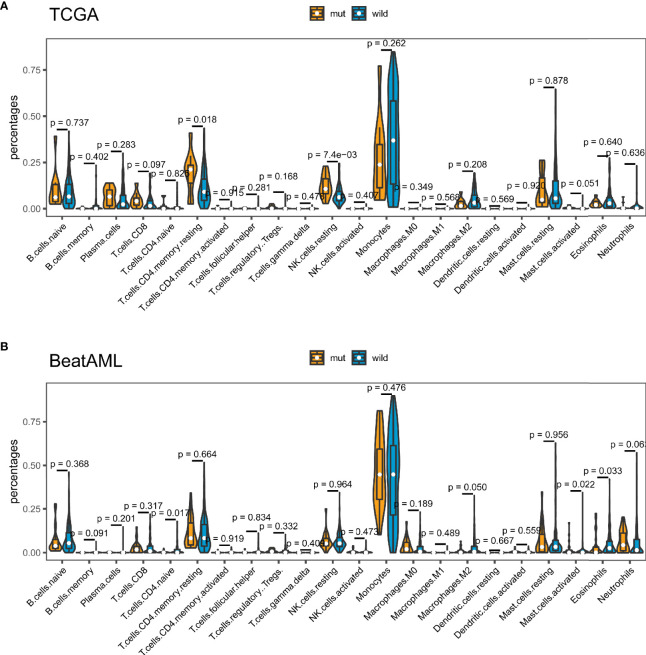
*TP53* mutation was related to tumor-infiltrating immune cells. Violin plots displayed the differences in the immune cell distribution between *TP53* mutant group and *TP53* wild-type group in The Cancer Genome Atlas (TCGA) cohort **(A)** and BeatAML cohort **(B)**.

**Figure 7:**
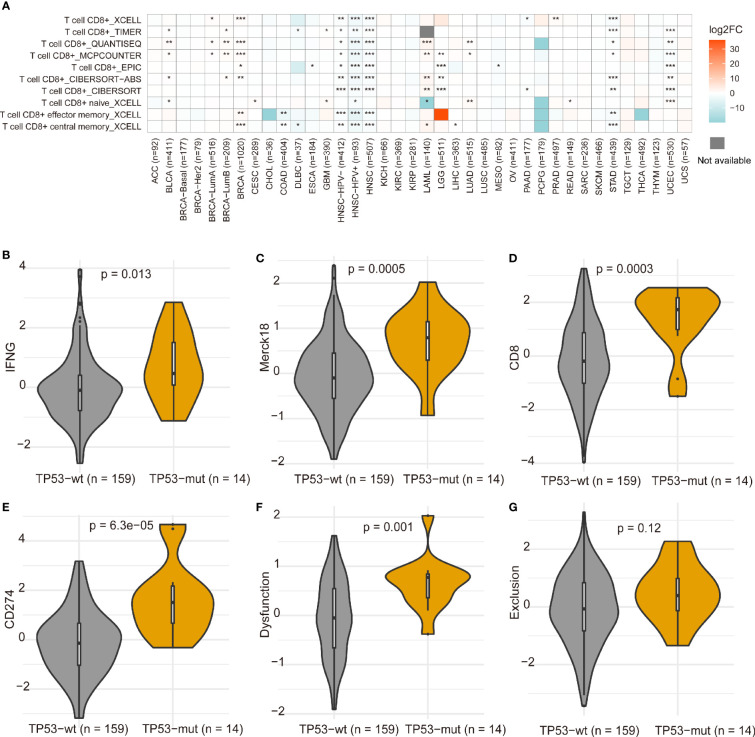
Differences of CD8+ T cells between *TP53* mutation and wild-type in pan-cancer **(A)**. *p < 0.05, **p < 0.01, ***p < 0.001. The differences of gene expression signatures calculated using TIDE between *TP53* mutant and non-mutant groups **(B–G)**. The immunotherapy clinical response prediction performance of TP53 mutant group and TP53 wild-type group was compared with those of the following biomarkers: IFGN, Merck18, CD8, CD274, dysfunction, and exclusion.

Tumor-infiltrating immune cells can illustrate either tumor-suppressive or tumor-promoting effects. We evaluated the relationship between *TP53* mutation and tumor-infiltrating immune cells in AML microenvironment using CIBERSORT algorithm. As shown in **Figure 6A**, we observed that resting memory CD4 T cells and resting NK cells were more enriched in *TP53* mutant type group from TCGA dataset (p = 0.018 and p < 0.001, respectively), while activated mast cells showed a tendency to be enriched in wild-type group (p = 0.051). Nevertheless, the *TP53* wild-type group had more naive CD4 T cells, activated mast cells, and eosinophils from BeatAML dataset (p = 0.017, p = 0.022, and p = 0.033, respectively) (**Figure 6B**). Besides, M2 macrophages had the tendency to be enriched in *TP53* wild-type group than *TP53* mutant group (p = 0.05), which would hamper the immune antitumor effect. Also, the *FLT3* mutant group had more M2 macrophages and eosinophils in both datasets (**Figure S6**). Likewise, eosinophils were more enriched in *NPM1* mutant group than *NPM1* wild-type group across two distinct datasets (**Figure S7**). Differences of CD8+ T cells between *TP53* mutated and wild-type were validated by multiple algorithms in pan-cancer, and CD8+ T cells were highly infiltrated in the *TP53* mutant group of TCGA AML dataset (**Figure 7A**). In immunotherapy prediction analysis with the online tool TIDE, *TP53* mutation was highly related to IFGN, Merck18, CD8, CD274, and dysfunction except for exclusion, which were widely used immunotherapy indicators (**Figures 7B** **–G**). *TET2*, *RUNX1*, *ASXL1*, *U2AF1*, and *PHF6* mutations presented increased TMB in both TCGA and BeatAML cohorts. We also evaluated the differences of CD8+ T cells of these gene mutated and wild–type groups; interestingly enough, no statistical difference was found in AML and other cancers (**Figure S8**).

### Enrichment Pathway Analysis of *TP53* Mutation

**Figure 8:**
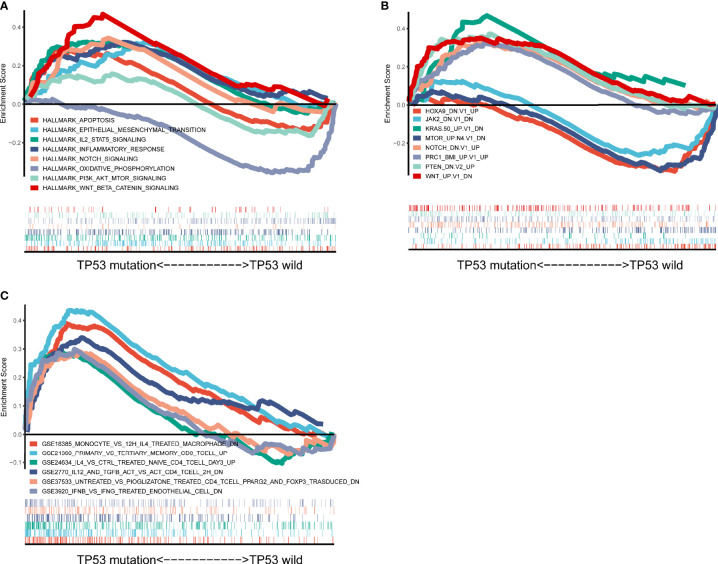
Significantly enriched pathways associated with *TP53* mutation. Gene set enrichment analyses were carried out with The Cancer Genome Atlas (TCGA) based on analysis of Hallmark gene sets **(A)**, curated gene sets **(B)**, and immunologic signature gene sets **(C)**.

To further explore the difference between *TP53* mutant and wild-type groups, we performed GSEA based on RNA sequencing (RNA-seq) data from TCGA, which showed a prominent enrichment in *TP53* mutant group of signatures related to wnt beta catenin signaling, IL2 signal transducer and activator of transcription (stat)5 signaling, notch signaling, and inflammatory response (**Figure 8A**). These findings indicated that samples with *TP53* mutation upregulated signaling pathways involved in the immune system. Also, our results confirmed and extended the above findings (**Figure 8B**): 1) A set of genes that caused characteristic downregulation after *KRAS* overexpression in the four epithelial cell lines was significantly enriched in the *TP53* mutant group. 2) *TP53* mutant group showed significant enrichment of genes downregulated in C57MG cells (mammary epithelium) by overexpression of *WNT1* gene. 3) *TP53* mutant group revealed enrichment of genes upregulated in HCT116 cells (colon carcinoma) upon knockdown of *PTEN* by RNAi. Together, these results further demonstrated that *TP53* mutation was associated with distinct molecular features in AML. For the C7 immunologic collection, the *TP53* mutated group had strong enrichment in genes downregulated in monocytes compared to macrophages treated with interleukin (IL)-4 (**Figure 8C**).

## Discussion

*TP53*, located on chromosome 17p13.1, encodes a 393-amino acid phosphoprotein and acts as a transcription factor with pivotal tumor suppressor functionality (25). *TP53* mutations are observed in about half of all cancers (26). However, compared with solid tumors, *TP53* mutations are rare and closely associated with AML with complex karyotype (13, 27, 28). Our findings underlined the poor prognostic impact of *TP53* mutation in AML patients. This observation was largely consistent with published data (18). AML patients with *TP53* mutations have dismal outcomes, with median OS of 5–9 months and complete remission (CR) rates of 20%–40% (18, 29–31). The mutation frequency of *TP53* is much higher in therapy-related AML (t-AML) than in *de novo* AML. The P53 protein contains three key regions: the N-terminal region, the central DNA-binding domain (DBD), and the C-terminal region (32). Meanwhile, most *TP53* mutations fall within the DBD (32).

High TMB has been proposed as a leading candidate biomarker for response to immunotherapy based on the underlying assumption that tumor mutations will generate antigenic peptides, allowing for enhanced immunogenicity (33, 34). Compared to highly mutated solid tumors, AML has low mutational burdens, with the exception of cases harboring mutations involving DNA mismatch repair genes (35). Nonetheless, profiling of AML patients relapsing after allogeneic hematopoietic stem cell transplantation displayed that T-cell exhaustion was a crucial contributor to failure of the leukemia relapse (36, 37), indicating that ICI can also be an appealing strategy for treatment of these patients. *TP53* mutations showed higher TMB levels in TCGA cohort of this study. We speculated that *TP53* mutation might participate in the immune response.

The biology of a tumor can only be understood by tumor-intrinsic alterations and the tumor microenvironment, especially the immune cells (38). Immune cells in the tumor immune microenvironment play a critical role in tumorigenesis, and tumor-related immune cells can act as antagonizing or promoting tumors. Apart from the recognition of the crucial role of the immune system in oncogenesis, tumor progression, and therapy response, more and more attention has been attracted by the potential predictive role of immune infiltration. For example, cytotoxic CD8+ T cells act as an indicator of favorable outcome in colorectal, ovarian, and esophageal cancer, whereas immunosuppressive cells such as Tregs and M2-polarized tumor-associated macrophages (TAMs) predict worse prognosis in several cancer types (39). Therefore, we need to investigate and understand the overall characteristics of the tumor immune microenvironment. In our study, *TP53* mutant group exhibited higher CD8+ T cell infiltration compared with *TP53* wild-type group in AML. However, no difference was found between *TET2*, *RUNX1*, *ASXL1*, *U2AF1*, and *PHF6* mutant and wild-type groups in AML and other cancers. And *TP53* mutation plays a key role in activating genes involved in immune responses and inflammation (40). Also, resting memory CD4 T cells and resting NK cells were found to be enriched in *TP53* mutant group, while naive CD4 T cells, activated mast cells, and eosinophils were more increased in *TP53* wild-type group. It is reported that resting memory CD4+ T cells are often related to prognosis of malignant tumor diseases, such as head and neck squamous cell carcinoma (41) and bladder cancer (42). Compared to naive T cells, memory CD4+ T cells show higher numbers, display distinct trafficking behaviors, and generally have faster effector function following reinfection (43). And NK cells were involved in tumor immune surveillance (44). Hsu et al. (45) proposed that blocking PD-1/PD-L1 may activate NK cells that were indispensable for the treatment effect of these therapies. Mast cells had higher proportions in *TP53* wild-type group than *TP53* mutant group in this study. It was reported that tumor-associated mast cells (TAMCs) were components of the microenvironment of hematologic human tumors (46). And mast cells owed both tumor-promoting and tumor-suppressive roles, which depend on local stromal conditions (47).

Our study revealed that IL-2 stat5 signaling was enriched in *TP53* mutant group. IL-2 was involved in Treg-mediated immunosuppressive mechanisms, and transforming growth factor (TGF)-β1 activates STAT5 binding to the promoter of Foxp3 to induce the differentiation of Tregs *via* IL-2 (48, 49). It indicated that *TP53* mutation may upregulate signaling pathways involved in the immune system. Further research is needed to study the underlying mechanism. *TP53* mutant groups showed significant enrichment of genes downregulated in C57MG cells (mammary epithelium) by overexpression of *WNT1* gene, and a prominent enrichment of signatures related to wnt beta catenin signaling in the present study. Deregulation of Wnt/β-catenin signaling is well-connected with AML initiation and progression and important to leukemia stem cell (LSC) self-renewal and survival (50, 51). Our results were further evidence that *TP53* mutation was associated with cancer hallmarks.

We analyzed the somatic mutation landscape of 164 AML samples from TCGA dataset and 558 AML patients from BeatAML dataset. We discovered that *TP53* was frequently mutated in both TCGA and BeatAML cohorts, and its mutation displayed exclusiveness with other common mutated genes and was associated with poor prognosis, TMB, and immune microenvironment. Further experiments are required to verify the relationship between *TP53* and TMB and immune infiltration in AML. Also, in view of the fact that CD8+ T cells showed high infiltration in the *TP53* mutant group of LAML, our findings therefore inspire further studies of T cell–targeting immunotherapeutic approaches in *TP53*-mutated AML. We consider co-cultivating T cells with *TP53* mutant and wild-type cells, then use flow cytometry, Cell Counting Kit-8 (CCK8), and other experiments to detect cytokines, cell proliferation and invasion, and so on in the following research to further validate these findings.

## Data Availability Statement

The original contributions presented in the study are included in the article/**Supplementary Material**. Further inquiries can be directed to the corresponding authors.

## Author Contributions

JQ conceived and designed the research. Z-JX and J-CM collected and analyzed data. X-MW, YJ, and P-HX prepared the figures and performed data analysis. X-MW and Z-JX drafted the article. JL and WQ participated in study supervision. All authors contributed to the article and approved the submitted version.

## Funding

This study was supported by the National Natural Science Foundation of China (81970118, 81900163), Medical Innovation Team of Jiangsu Province (CXTDB2017002), Zhenjiang Clinical Research Center of Hematology (SS2018009), Social Development Foundation of Zhenjiang (SH2019065, SH2019067), and Scientific Research Project of The Fifth 169 Project of Zhenjiang (21).

## Conflict of Interest

The authors declare that the research was conducted in the absence of any commercial or financial relationships that could be construed as a potential conflict of interest.

## Publisher’s Note

All claims expressed in this article are solely those of the authors and do not necessarily represent those of their affiliated organizations, or those of the publisher, the editors and the reviewers. Any product that may be evaluated in this article, or claim that may be made by its manufacturer, is not guaranteed or endorsed by the publisher.

## Supplementary Material

Supplementary Figure 1 The most frequently mutated genes in AML. Venn diagram showing the overlap of frequently mutated genes covered by both TCGA and BeatAML cohorts.

Supplementary Figure 2 Association of mutated genes with prognosis by Kaplan–Meier analysis. **(A)** *DNMT3A* mutation and *RUNX1* mutation were significantly correlated with poor outcome in TCGA cohort. **(B)** Kaplan-Meier survival analysis classified by *TET2* and *U2AF1* mutation status in BeatAML cohort.

Supplementary Figure 3 Gene mutations were associated with TMB. **(A, B)** The range of TMB among mutated genes in TCGA **(A)** and BeatAML **(B)** cohorts. **(C)** Most gene mutations were associated with a higher TMB in BeatAML cohort. *p < 0.05, **p < 0.01, ***p < 0.001.

Supplementary Figure 4 The association of *FLT3* and *NPM1* mutations with TMB in both TCGA and BeatAML cohorts.

Supplementary Figure 5 Relationship between *TP53* mutation status and *TP53* mRNA expression level in BeatAML database.

Supplementary Figure 6 Correlation of *FLT3* mutations with tumor-infiltrating immune cells in both TCGA and BeatAML cohorts.

Supplementary Figure 7 The association of *NPM1* mutations with tumor-infiltrating immune cells in both TCGA and BeatAML cohorts.

Supplementary Figure 8 Differences of CD8+ T cells between *TET2*, *RUNX1*, *ASXL1*, *U2AF1* and *PHF6* mutant and wild groups in pan-cancer calculated using TIDE.

Supplementary Tables 1 and 2 Summary of the maf file, which displays number of variants in each gene of TCGA and BeatAML cohorts.

Supplementary Tables 3 and 4 Original data with somatic genomic interactions from TCGA and BeatAML cohorts **(Figures 2C, D)**.

Supplementary Tables 5 and 6 Original data used for Circos plot diagram **(Figures 2A, B)** from TCGA and BeatAML cohorts.
