# Supplementary figures and images for: Association Analyses of TP53 Mutation With Prognosis, Tumor Mutational Burden, and Immunological Features in Acute Myeloid Leukemia
Source: Front Immunol. 2021 Oct 21;12:717527. doi: 10.3389/fimmu.2021.717527 (PMC8566372; doi:10.3389/fimmu.2021.717527)

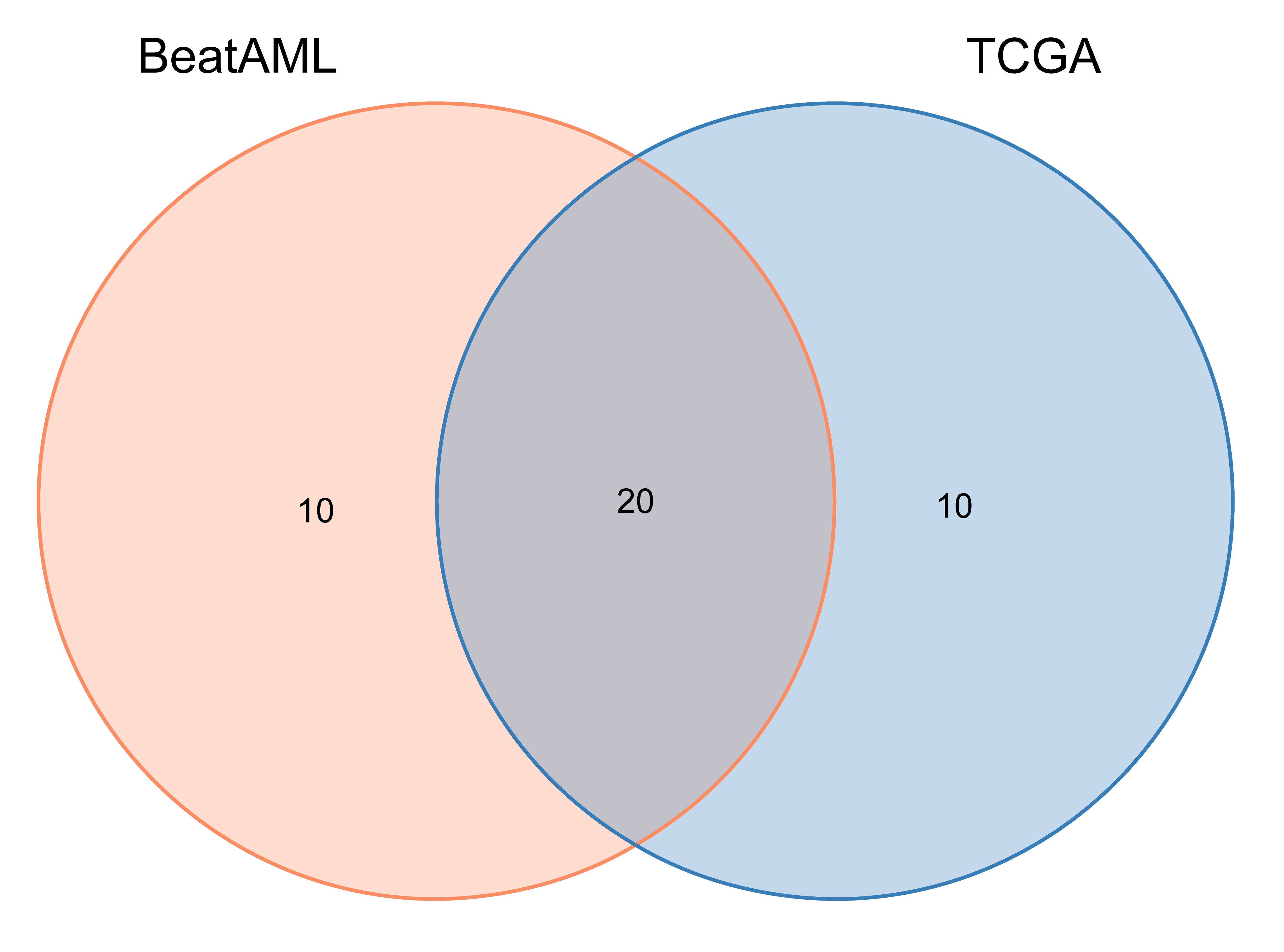

Supplement: Supplementary Figure 1 — The most frequently mutated genes in AML. Venn diagram showing the overlap of frequently mutated genes covered by both TCGA and BeatAML cohorts. [file Image_1.tif]

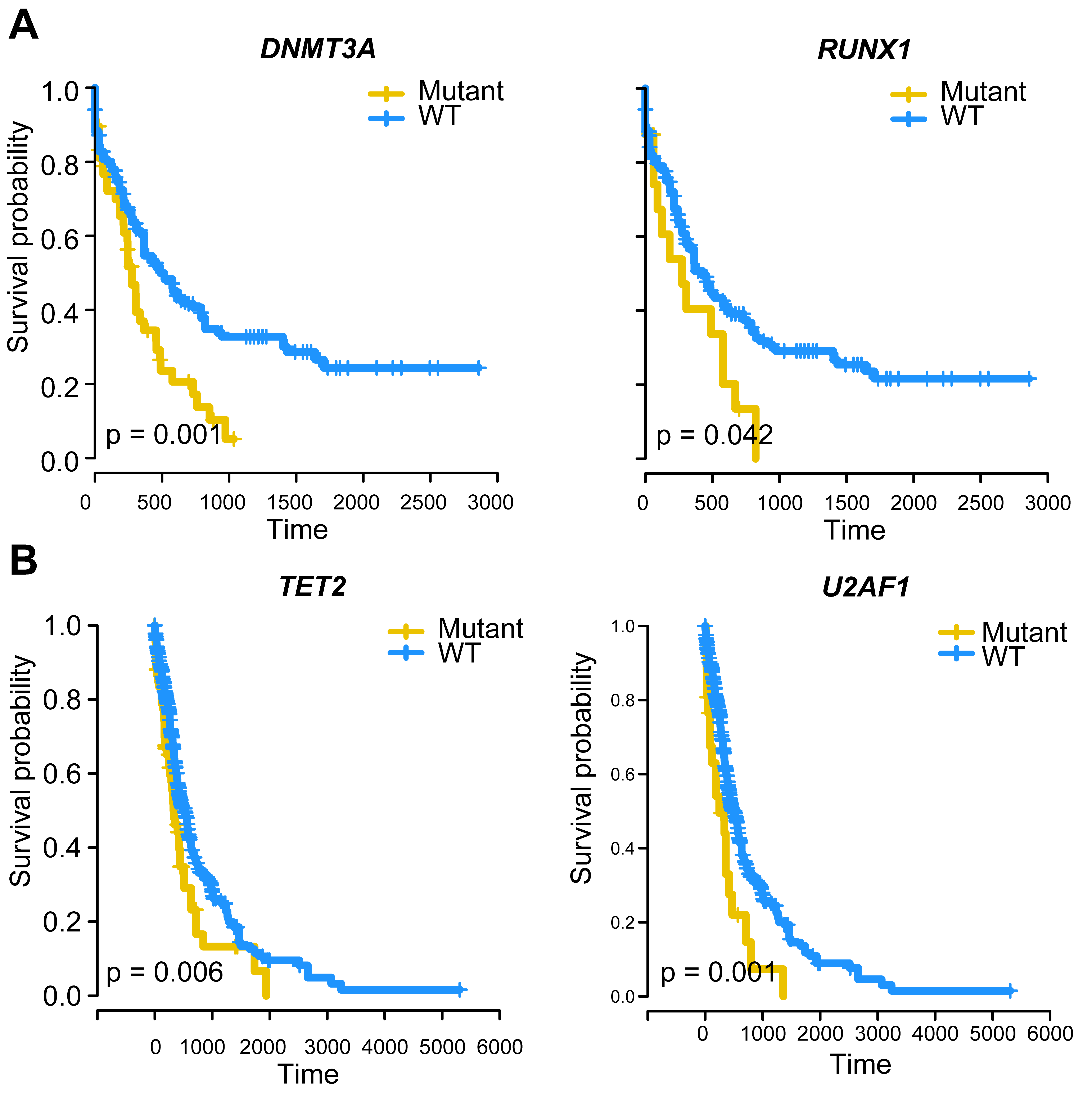

Supplement: Supplementary Figure 2 — Association of mutated genes with prognosis by Kaplan–Meier analysis. (A) DNMT3A mutation and RUNX1 mutation were significantly correlated with poor outcome in TCGA cohort. (B) Kaplan-Meier survival analysis classified by TET2 and U2AF1 mutation status in BeatAML cohort. [file Image_2.tif]

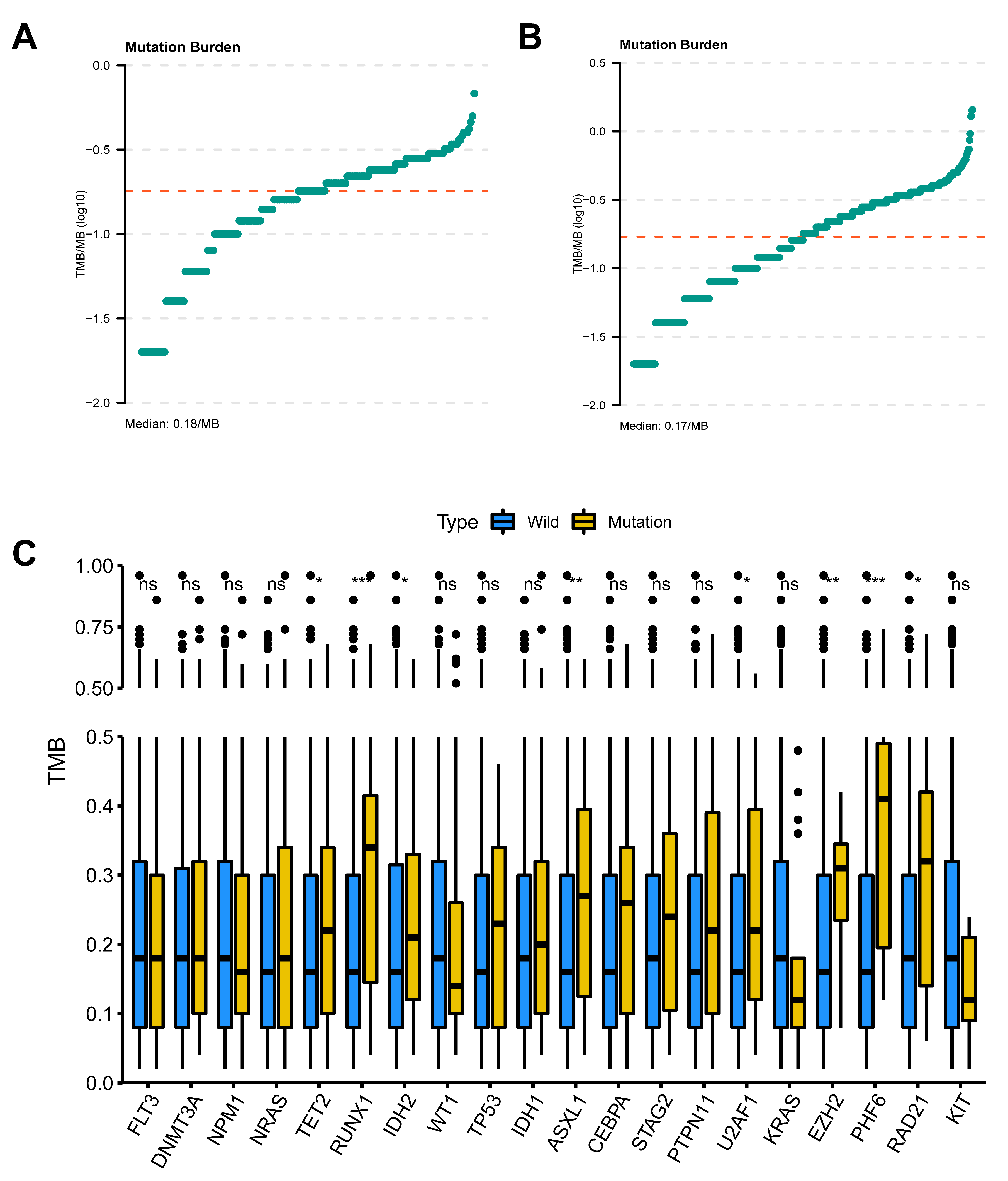

Supplement: Supplementary Figure 3 — Gene mutations were associated with TMB. (A, B) The range of TMB among mutated genes in TCGA (A) and BeatAML (B) cohorts. (C) Most gene mutations were associated with a higher TMB in BeatAML cohort. *p < 0.05, **p < 0.01, ***p < 0.001. [file Image_3.tif]

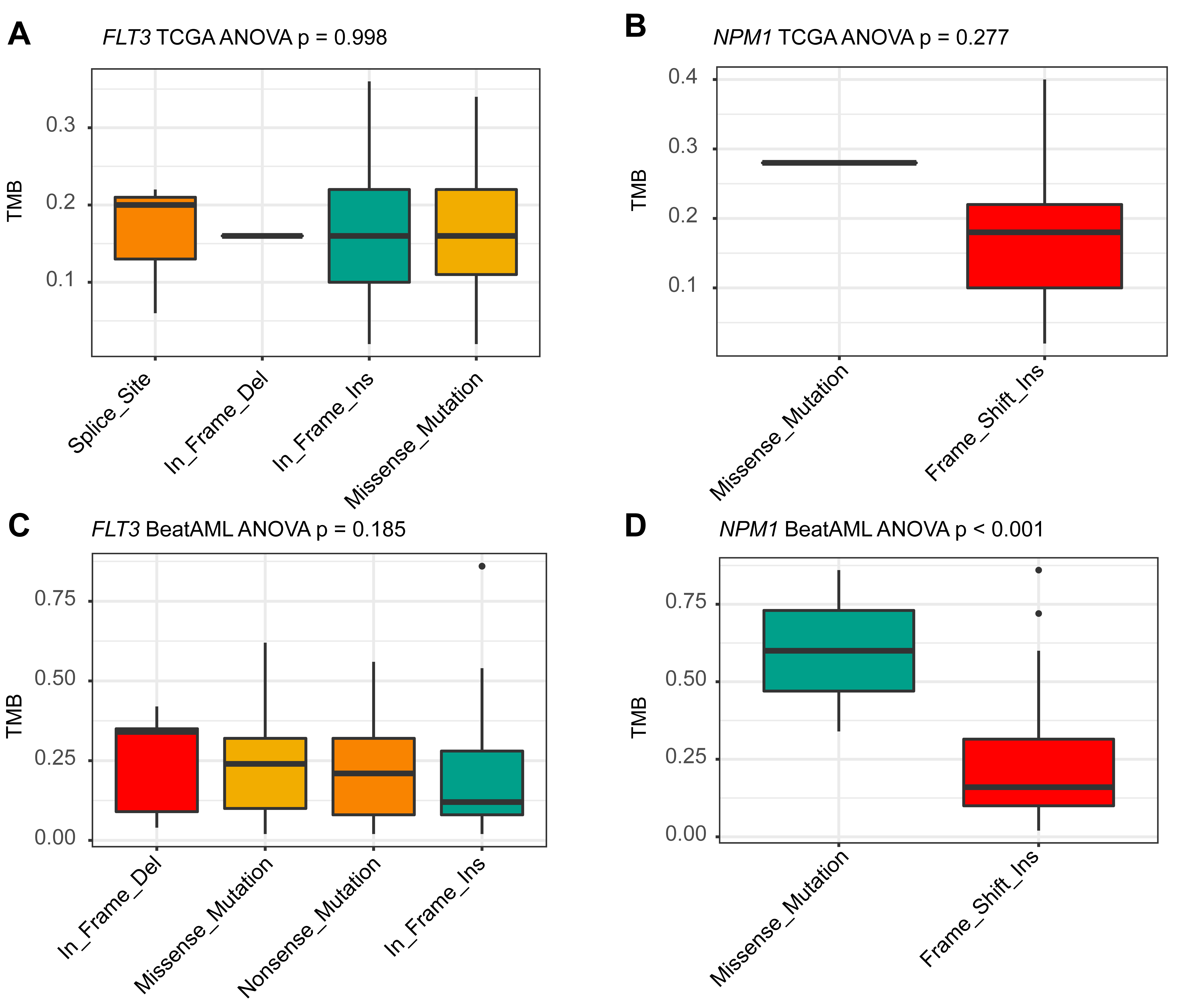

Supplement: Supplementary Figure 4 — The association of FLT3 and NPM1 mutations with TMB in both TCGA and BeatAML cohorts. [file Image_4.tif]

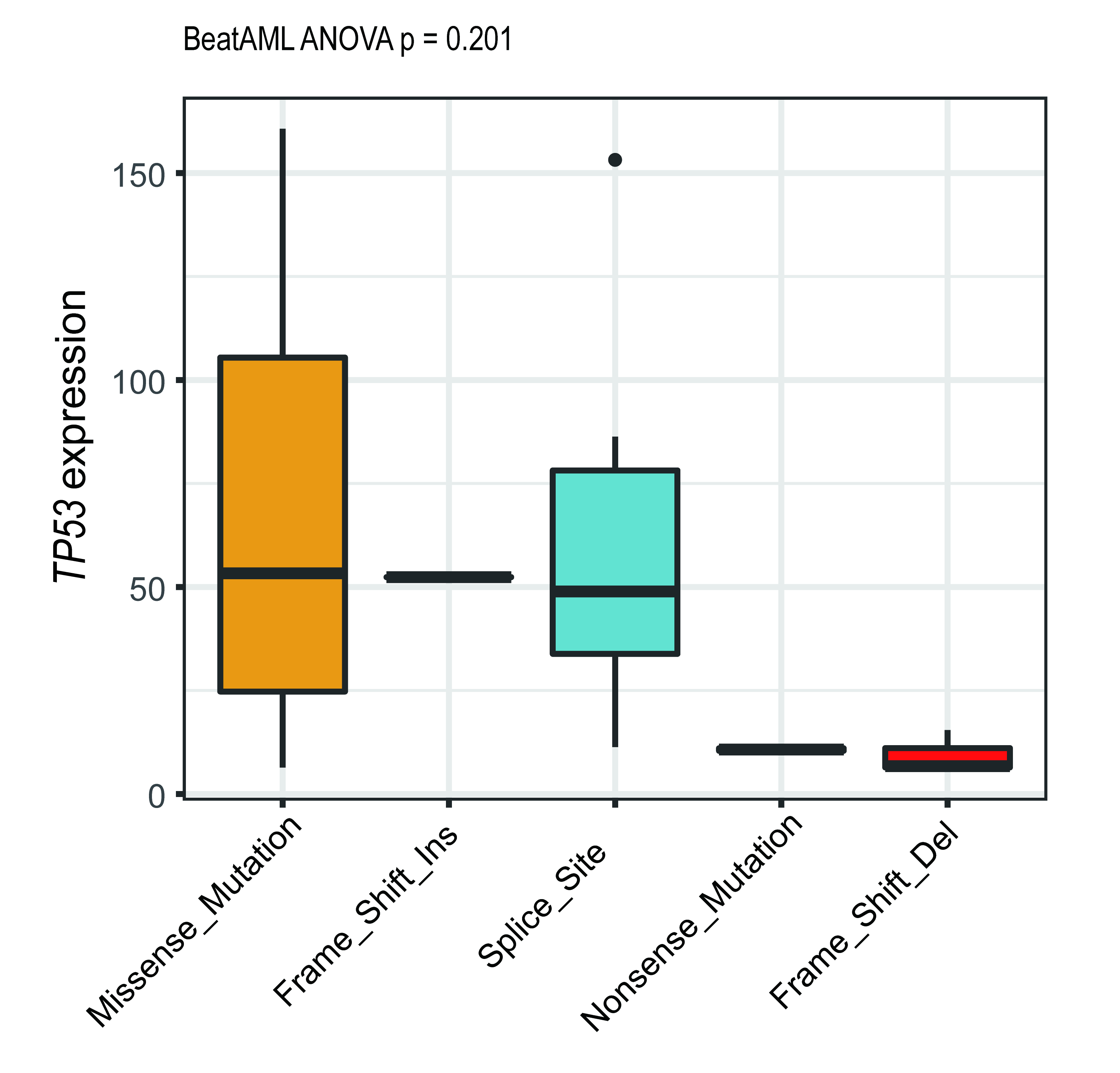

Supplement: Supplementary Figure 5 — Relationship between TP53 mutation status and TP53 mRNA expression level in BeatAML database. [file Image_5.tif]

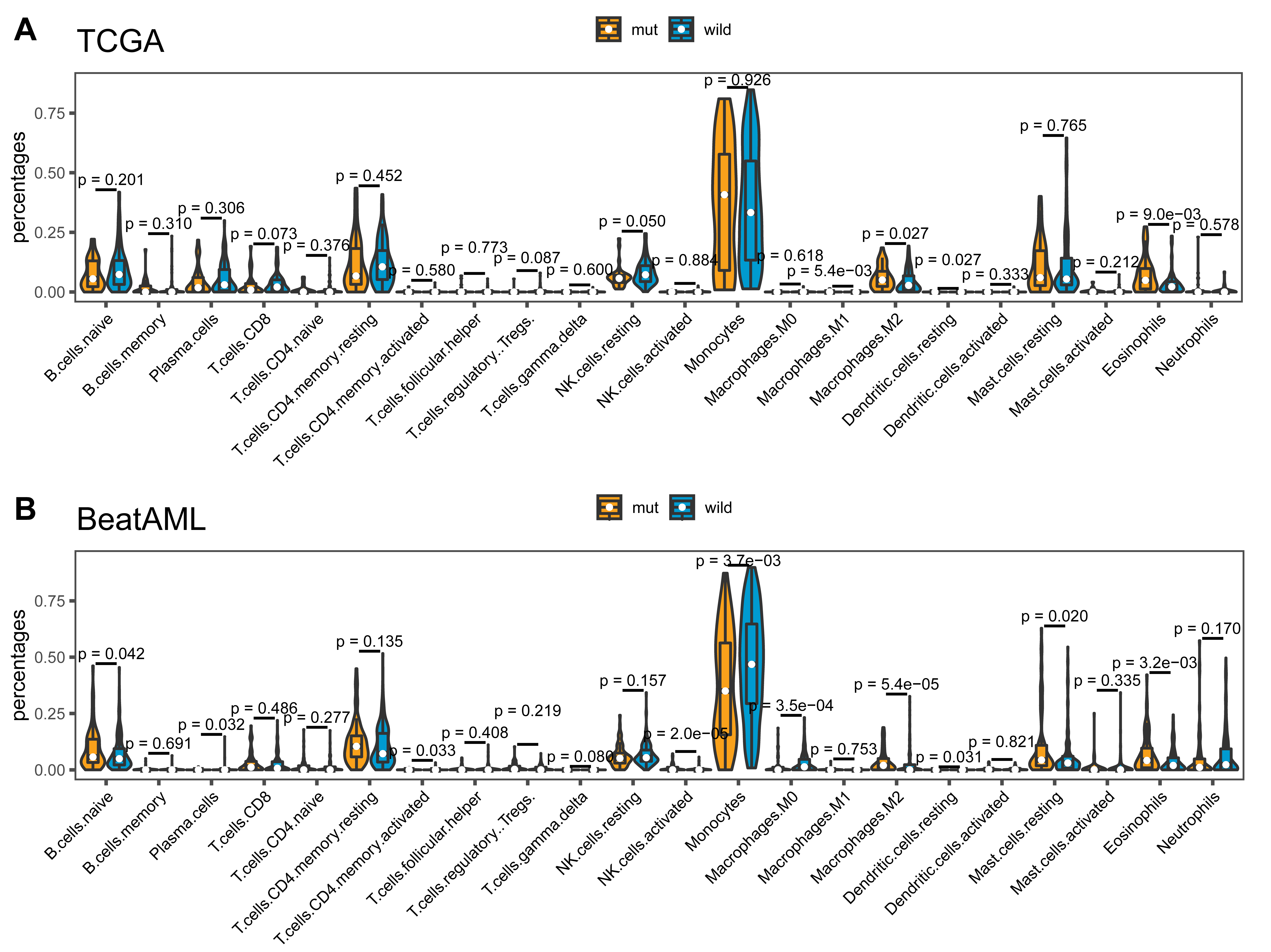

Supplement: Supplementary Figure 6 — Correlation of FLT3 mutations with tumor-infiltrating immune cells in both TCGA and BeatAML cohorts. [file Image_6.tif]

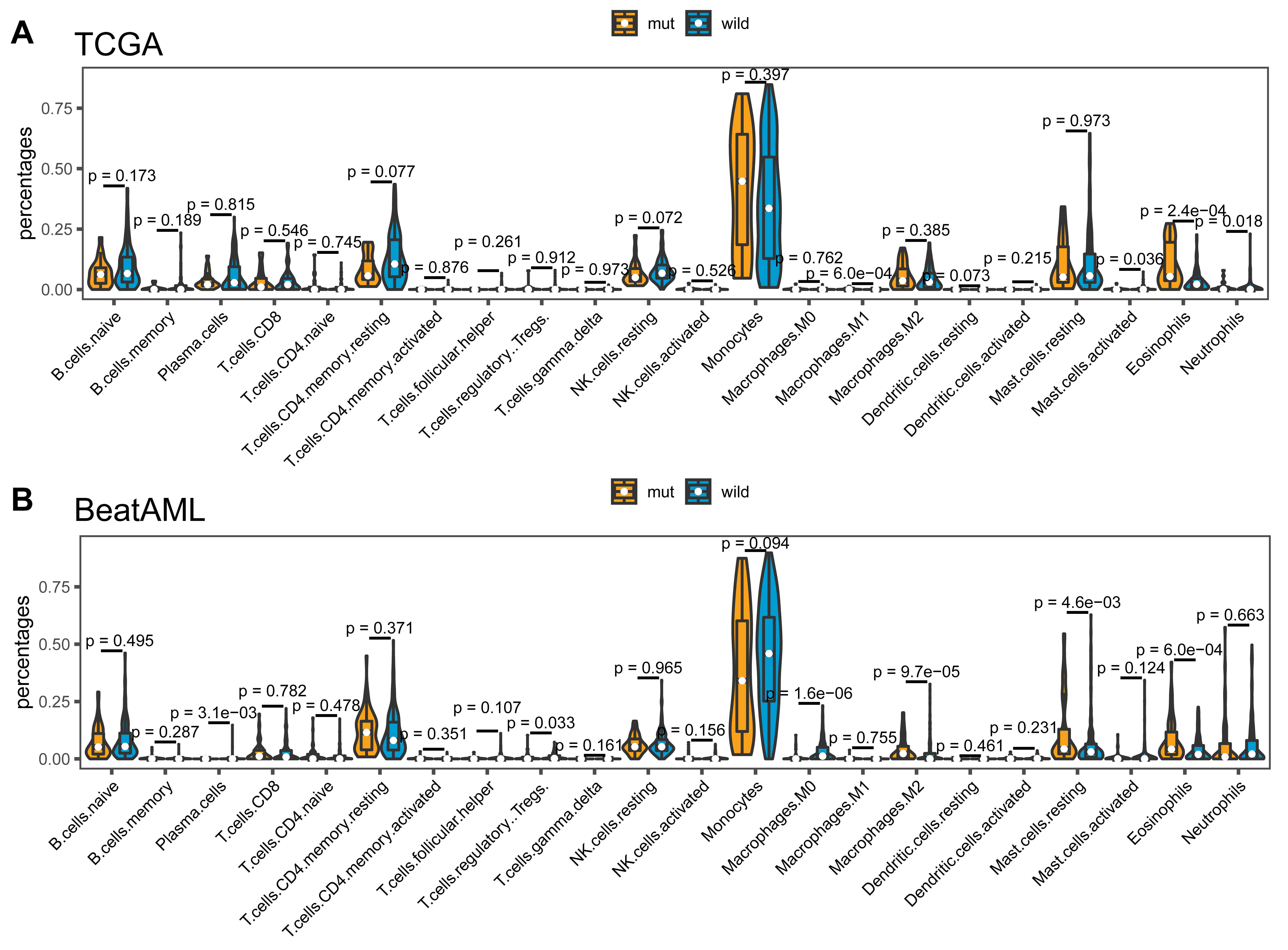

Supplement: Supplementary Figure 7 — The association of NPM1 mutations with tumor-infiltrating immune cells in both TCGA and BeatAML cohorts. [file Image_7.tif]

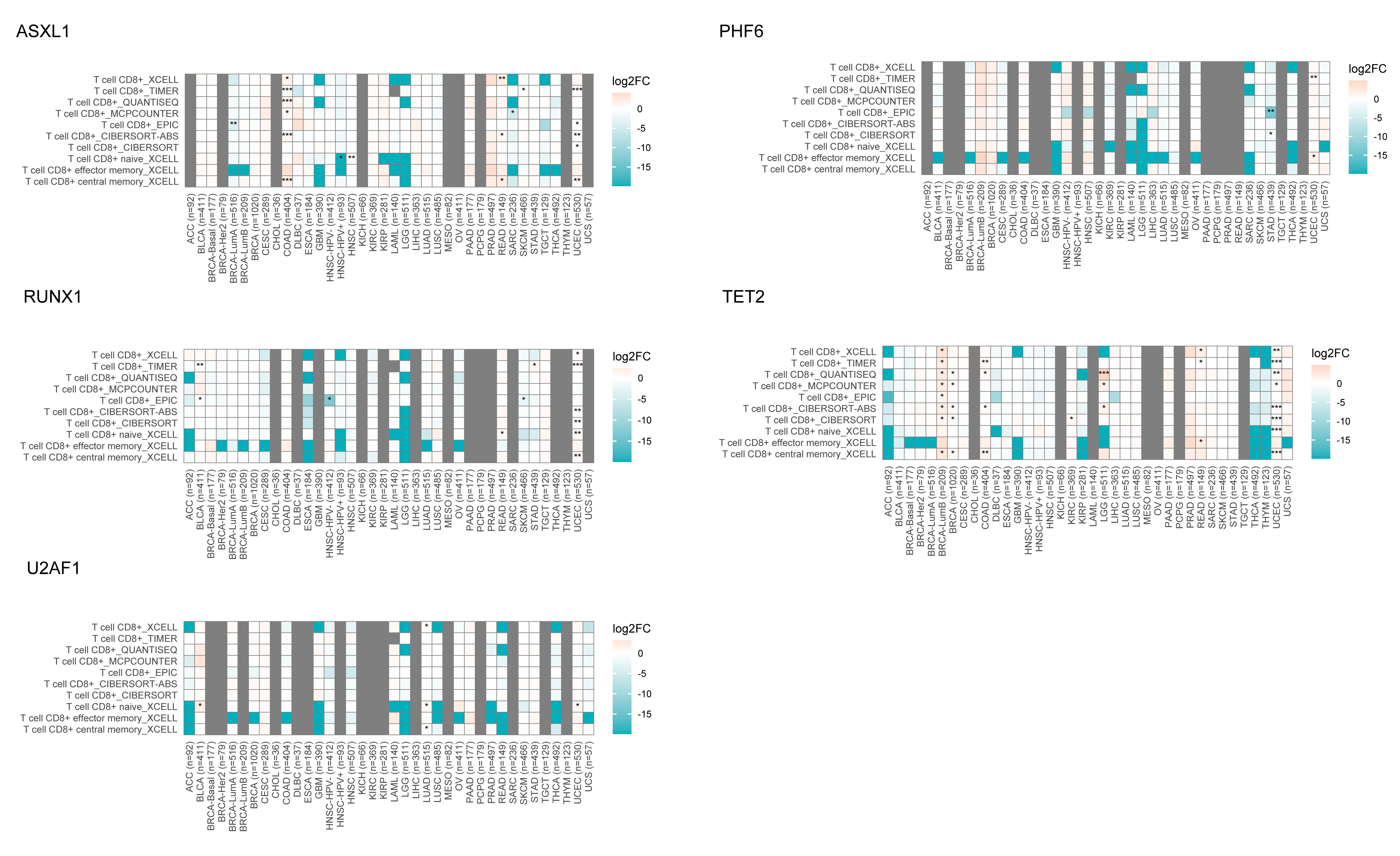

Supplement: Supplementary Figure 8 — Differences of CD8+ T cells between TET2, RUNX1, ASXL1, U2AF1 and PHF6 mutant and wild groups in pan-cancer calculated using TIDE. [file Image_8.tif]
